# Left Ventricular Pseudoaneurysm Secondary to Mitral Valve Endocarditis

**DOI:** 10.5935/abc.20150071

**Published:** 2015-09

**Authors:** Bruno Bochard-Villanueva, Jordi Estornell-Erill

**Affiliations:** 1 Departamento de Cardiologia, Consórcio Hospital Geral Universitário de Valência - Spain; 2 Unidade de Imagem Cardíaca ERESA, Consórcio Hospital Geral Universitário de Valência – Spain

**Keywords:** Ventricular Dysfunction, Left / complications, Heart Failure, Heart Valve Diseases, Endocarditis, Bacterial, Echocardiography, Transesophageal, Tomography, X-Ray Computed

A follow-up transesophageal echocardiography (TEE) was performed on a 76-year-old woman with a recent history of mitral valve endocarditis after 4 weeks of antibiotic treatment. TEE showed a pulsatile perivalvular echo-free space of 32 × 23 mm with a narrow orifice which communicated to the left ventricle at the posterior mitral subannular position consistent with the pseudoaneurysm (Panel A). The real-time three-dimensional TEE allowed us to see its relationship with the neighboring structures (Panels B and C). Subsequently, a coronary CT angiogram confirmed these findings and revealed no significant coronary stenosis (Panels D and E). Therefore, surgery was indicated and a bovine pericardium patch was implanted with good results.

## Figures and Tables

**Figure 1:**
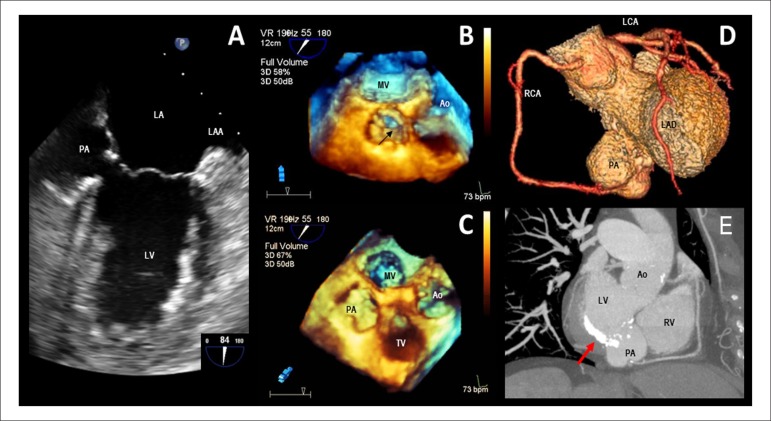
A. Transesophageal echocardiography (TEE) showing a pulsatile perivalvular echo-free space of 32 × 23 mm corresponding with a basal inferior pseudoaneurysm (PA). B, C. Real-time 3D TEE showing PA and its relationship with adjacent structures. D. A 3D volume-rendered cardiac CT angiogram showing PA and coronary arteries. E. Maximum intensity pixel projection reconstruction using a short axis view at the level of the mitral valve showing PA. Note the severe mitral annular calcification (arrow).

